# High-grade B-cell lymphoma presenting with bulbar palsy and Tapia syndrome: A case report

**DOI:** 10.1097/MD.0000000000050500

**Published:** 2026-09-04

**Authors:** Han Luo, Zengxia Zhao, Bo Liu

**Affiliations:** a Department of Neurology, Shenzhen Longhua District Central Hospital, Shenzhen, Guangdong, China.

**Keywords:** B symptoms, bulbar palsy, carotid artery stenosis, high-grade B-cell lymphoma, Tapia syndrome

## Abstract

**Rationale:**

Bulbar palsy and Tapia syndrome (ipsilateral recurrent laryngeal and hypoglossal nerve palsy) are rarely the initial manifestations of lymphoma. Motor neuron disease is often the primary consideration in such cases, potentially leading to diagnostic delay.

**Patient concerns:**

A 46-year-old male was admitted to the hospital on April 16, 2020, with the chief complaint of “dysphagia for 7 days.”

**Diagnosis:**

High-grade B-cell lymphoma.

**Interventions:**

The patient received swallowing function rehabilitation training after admission. On May 12, 2020, a biopsy of a painful mass in the right mandibular area was performed. On May 20, 2020, systemic anti-tumor therapy was administered based on immunohistochemistry and FISH results.

**Outcomes:**

At the 2-month follow-up, the patient showed further weight loss, extensive extranodal and multi-organ involvement, indicating a poor prognosis.

**Lessons:**

Lymphoma can present with isolated bulbar palsy and Tapia syndrome, mimicking motor neuron disease. Recognizing B symptoms and atypical neuroimaging findings is essential for early diagnosis. Furthermore, the etiology of carotid stenosis in lymphoma patients is often multifactorial, and treatment decisions must balance stroke prevention against bleeding and other tumor-related risks.

## 1. Introduction

Dysphagia and dysarthria are relatively common symptoms in neurological disorders, with motor neuron disease, myasthenia gravis, and cerebral infarction being their main etiologies. However, hematologic tumors such as lymphoma can directly invade the medulla oblongata and cranial nerves, manifesting as similar neurological deficit syndromes, which are highly susceptible to clinical misdiagnosis. This article reports the case of a 46-year-old male patient who presented initially with bulbar palsy and Tapia syndrome and was ultimately diagnosed with high-grade B-cell lymphoma. The clinical characteristics and early diagnostic clues of high-grade B-cell lymphoma are analyzed, aiming to improve the recognition of this disease.

## 2. Case description

### 2.1. Clinical presentation

A 46-year-old Han Chinese male was admitted to the hospital on April 16, 2020, with the chief complaint of “dysphagia for 7 days.” The patient developed dysphagia without obvious cause, accompanied by a choking sensation and mild difficulty in phonation, but without ptosis, diplopia, limb weakness, limb numbness, nausea, or vomiting. The symptoms were persistent, worsened after activity, and were slightly relieved by rest. He also reported occasional muscle fasciculations in the limbs of variable duration, with no muscle pain, joint pain, rash, or recurrent oral ulcers. During this period, his sleep was fair, appetite was poor, and bowel and bladder functions were normal; he had experienced a 5 kg weight loss over the preceding 2 months. His past medical history was unremarkable for hypertension, diabetes, coronary heart disease, tuberculosis, hepatitis, bacillary dysentery, typhoid fever, or relevant contact history, and he had no drug or food allergies. He smoked 10 cigarettes per day and did not consume alcohol. On admission, vital signs were as follows: T: 36.4°C, P: 107 bpm, R: 22 bpm, BP: 128/87 mm Hg. The abdomen was flat and soft, with no masses or guarding; the liver was not palpable, but the spleen was enlarged. Neurological examination showed that the patient was alert and responsive but dysarthric. Pupils were equal and round with reactive light reflexes; extraocular movements were full without nystagmus. Nasolabial folds were symmetric, and the mouth angle was midline on showing teeth. The uvula was midline; the right gag reflex was diminished; palatal elevation was symmetric bilaterally. Tongue deviation to the right, tongue atrophy, and tongue fasciculations were observed. There was wasting of the bilateral hand interossei and thenar muscles. Grade 5 muscle strength in all 4 limbs with normal and symmetric tone. Deep tendon reflexes were brisk throughout. Hoffmann and Rossolimo signs were positive bilaterally in the upper limbs; plantar responses were negative. No sensory deficits were detected. Finger-to-nose test, rapid alternating movements, and heel-knee-shin test were symmetric and accurate. The neck was supple, and Kernig sign was negative.

### 2.2. Assistant examinations

#### 2.2.1. Laboratory findings

Blood routine: Hb 100↓g/L, MCV 61.3↓fL, MCH 19.2↓pg, MCHC 313↓g/L. HsCRP: 40.2↑mg/L. Erythrocyte sedimentation rate: 40.0↑mm/h. TT3 3.16↑nmol/L, TT4 273.49↑nmol/L, FT3 11.050↑pmol/L. Thyroid peroxidase, thyrotropin receptor antibody, thyroglobulin antibody: normal. Rheumatologic screening: Cytoplasmic granular pattern 1:320 to 1:1000 positive. Lupus anticoagulant screening: TR 1.45 ↑, LA weakly positive. D-dimer: 0.70↑mg/L. Coagulation function: fibrinogen 5.44↑g/L. Lipids, cardiac enzymes, liver function, renal function, electrolytes, fasting glucose, homocysteine, human immunodeficiency virus antibody, syphilis antibody, etc: No significant abnormalities. Vasculitis screening, antiphospholipid antibody screening, immunoglobulins and complement, antinuclear antibody panel + anti-extractable nuclear antigen antibody profile: Normal. Neostigmine test: Negative.

#### 2.2.2. Neurophysiology

Electromyography (EMG): EMG of muscles innervated by cervical roots C5-8 and T1 bilaterally shows varying degrees of neurogenic changes, suggesting functional impairment of corresponding nerve roots or higher spinal cord segments. Left sternocleidomastoid EMG shows early neurogenic changes. EMG of right tibialis anterior, left vastus medialis, and right T10-11 paraspinal muscles is essentially normal. Repetitive nerve stimulation decrement test: negative.

#### 2.2.3. Imaging findings

**Figure 1.**
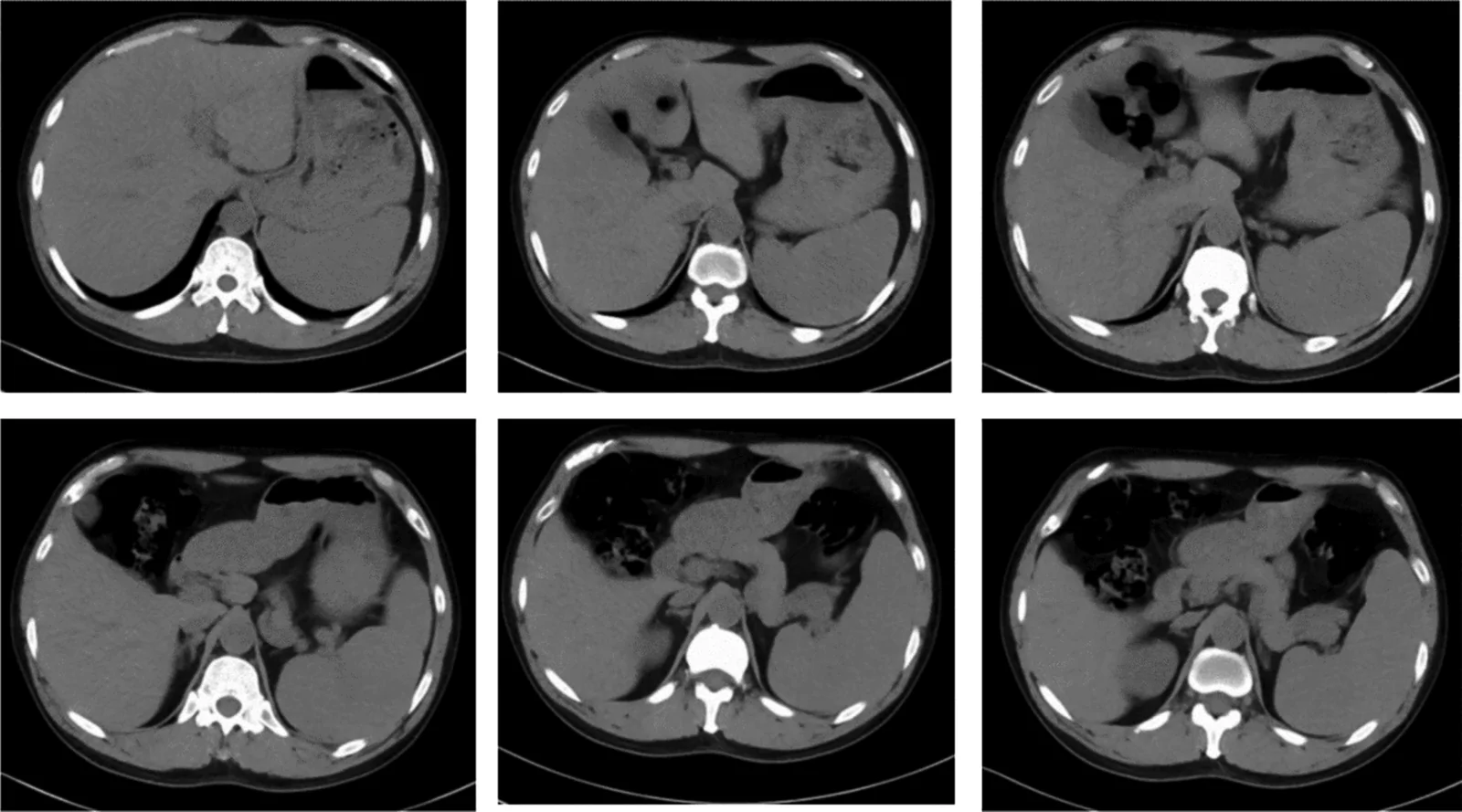
Chest CT incidentally shows splenomegaly. CT = computed tomography.

**Figure 2.**
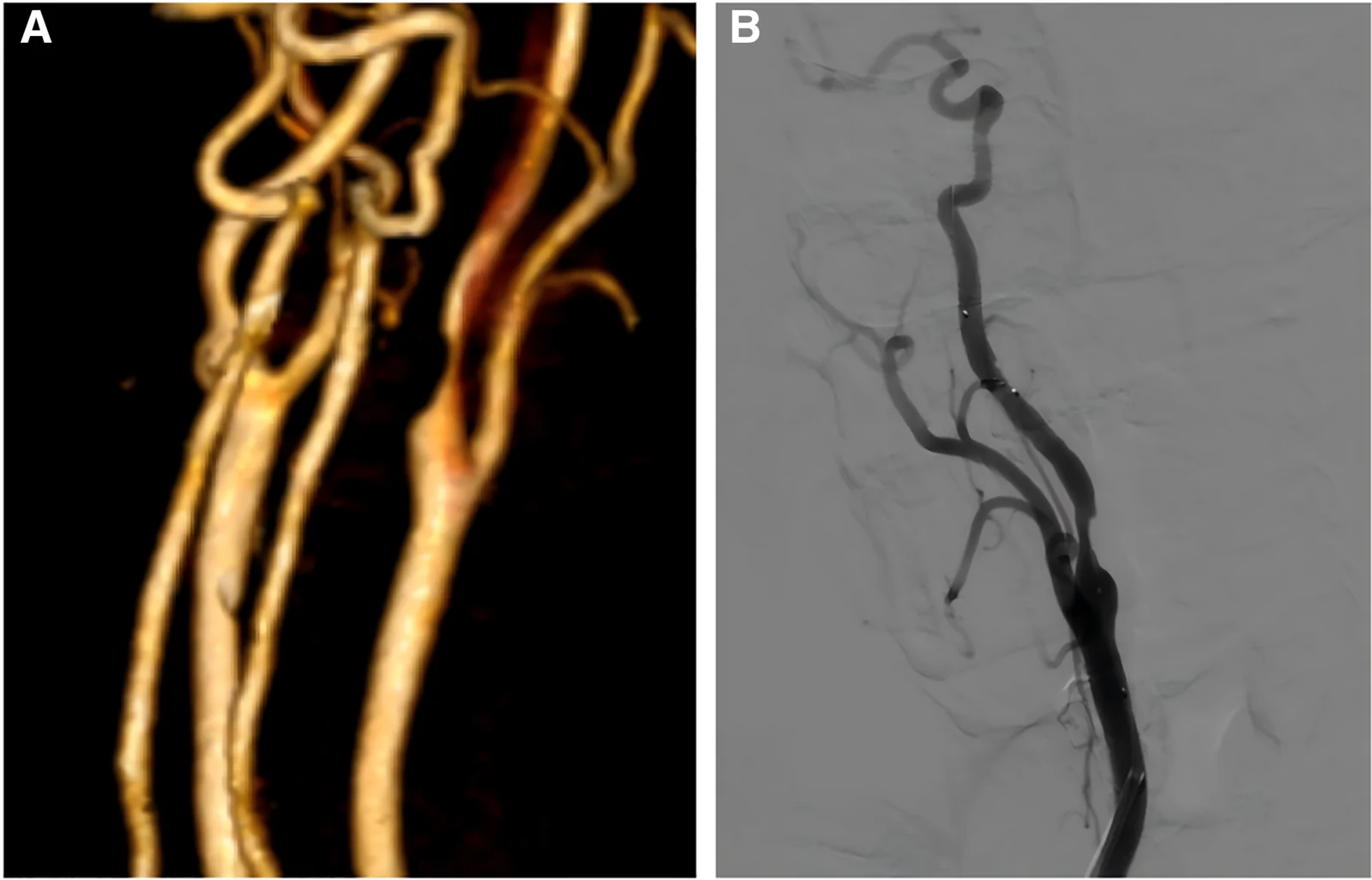
Head and neck CTA and cerebral angiography revealed stenosis of the right internal carotid artery. CTA = computed tomography angiography.

**Figure 3.**
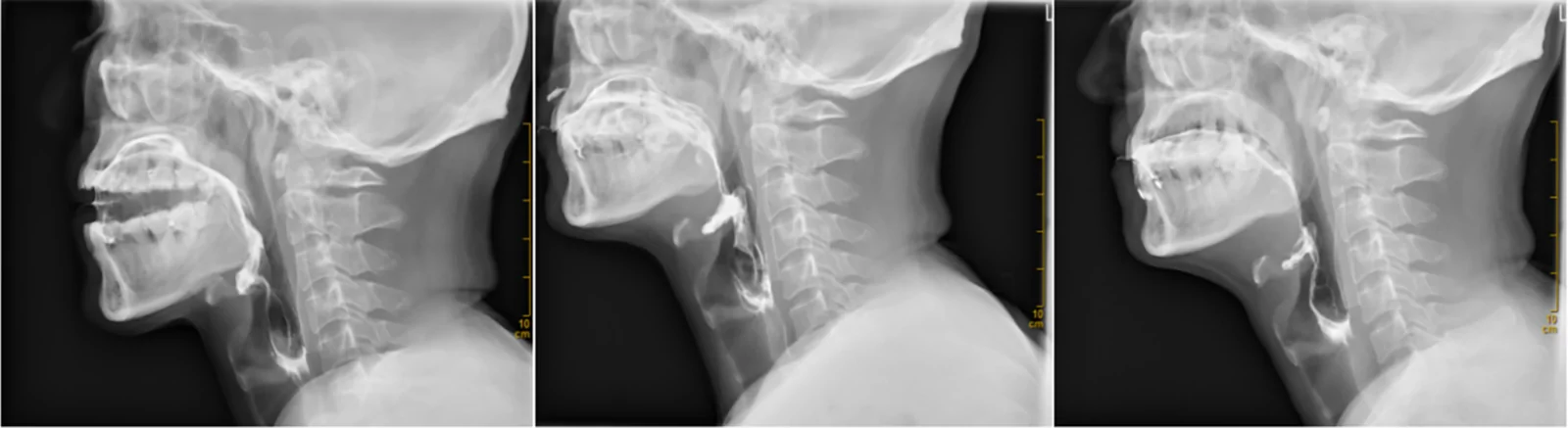
Barium swallow: Contrast retention in the valleculae and piriform sinuses.

**Figure 4.**
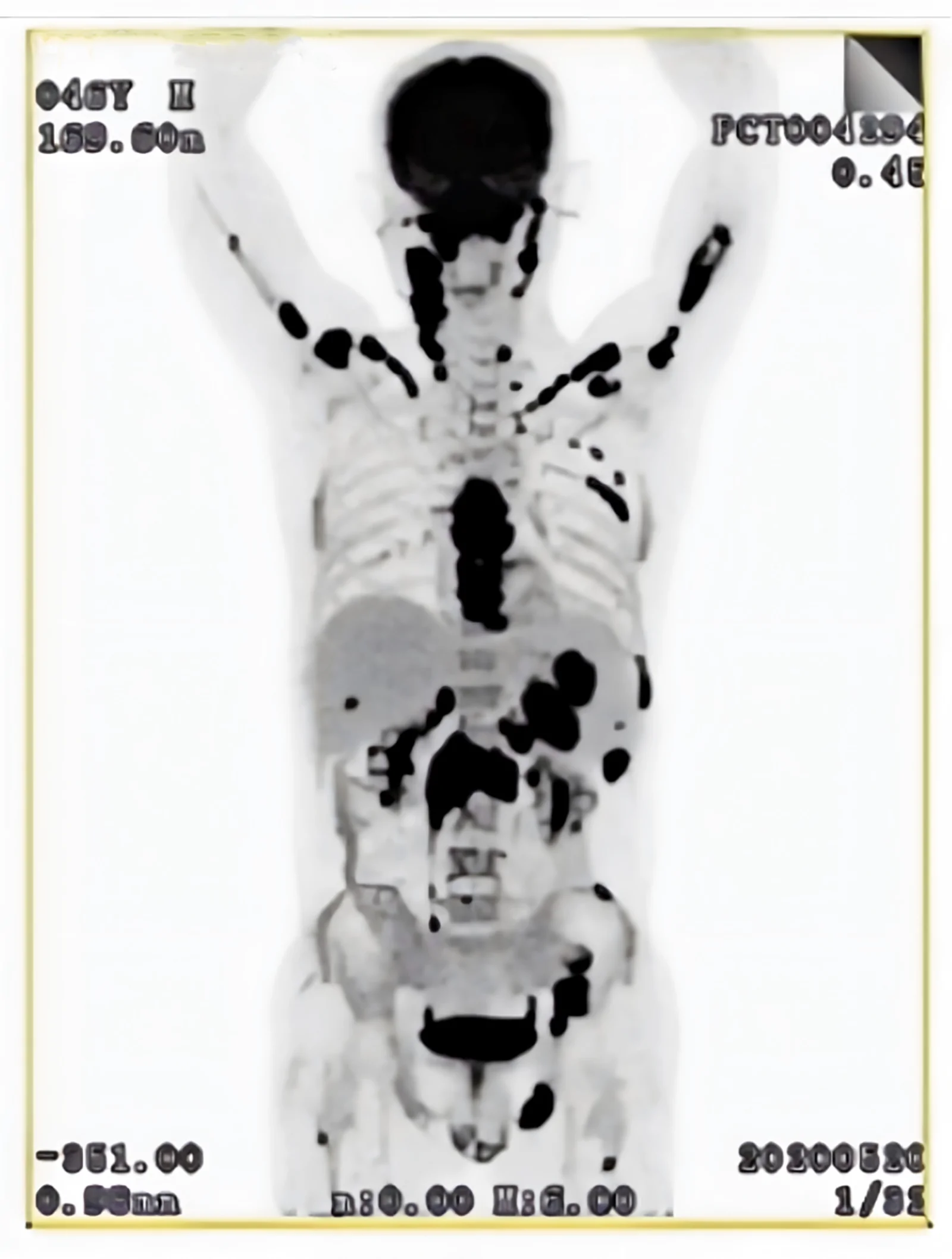
PET-CT of the patient. PET-CT = positron emission tomography-computed tomography.

Bilateral thyroid ultrasound: No abnormal findings in the thyroid. Upper and lower extremity arterial ultrasound: Atherosclerotic plaques in bilateral lower extremity arteries; bilateral upper extremity arteries show no significant abnormalities. Renal arteries and abdominal aorta: No significant abnormalities in bilateral renal artery blood flow; abdominal aortic atherosclerotic plaques without significant luminal stenosis. Echocardiography: ejection fraction 69%, no abnormalities in cardiac morphology and structure, normal left ventricular systolic and diastolic function. Transcranial Doppler: No significant abnormalities in the examined vessels. Carotid and vertebral artery ultrasound: Bilateral carotid artery atherosclerotic changes with plaque formation, left internal carotid artery origin stenosis < 50%, right internal carotid artery origin severe stenosis. Plaque formation at the origin of the right subclavian artery. Chest CT: Bilateral pulmonary emphysema with bullae formation; Fibrotic foci in the dorsal and posterior segments of both lower lobes; and Incidental splenomegaly (Fig. 1). Head and neck computed tomography angiography (CTA) (Fig. 2A): No significant abnormalities on head CTA; plaque at the origin of the right internal carotid artery with severe luminal stenosis. Localized soft plaque at the origin of the left internal carotid artery; incidental: nasopharyngeal soft tissue thickening. Cerebral angiography revealed stenosis of the right internal carotid artery (Fig. 2B). Brain magnetic resonance imaging: Few small ischemic foci adjacent to the anterior horns of the bilateral lateral ventricles. Incidental: Pan-sinusitis, bilateral maxillary sinus mucosal cysts; right inferior turbinate hypertrophy, nasopharyngeal soft tissue thickening, abnormal signal in the bilateral longus capitis muscles, clivus, and anterior part of the foramen magnum. Barium swallow (Fig. 3): Contrast retention in the valleculae and piriform sinuses. Submandibular mass: Multiple abnormal solid echoes in the right neck. On May 20, 2020, a positron emission tomography-computed tomography (PET-CT) scan was performed (Fig. 4): Large hypermetabolic lesion in the sphenoid sinus and nasopharynx, consistent with lymphoma involving the nasopharynx and sphenoid sinus. The tumor extends to invade the sphenoid body, greater and lesser wings of the sphenoid, bilateral pterygoid processes, medial and lateral plates of the right pterygoid, right medial pterygoid muscle, bilateral longus capitis muscles, bilateral petrous portions of the temporal bones, bilateral foramina lacerum, clivus and basiocciput, and the atlas, with possible invasion of the right temporal lobe base. Multiple hypermetabolic lesions in bilateral retropharyngeal spaces, right neck (levels IB, II–V), left neck (levels IB, II, VB), retroperitoneum, and splenic hilum, consistent with lymphoma involving multiple lymph nodes. Lymphoma focus adjacent to the gallbladder fossa in the right hepatic lobe; lymphoma foci in both kidneys; lymphoma foci in both adrenal glands; splenic mass-like lymphoma focus. Numerous hypermetabolic lesions throughout the skeleton, consistent with lymphoma involving multiple bone marrow sites, with L2 vertebral body complicated by compression fracture and paravertebral soft tissue mass invading the adjacent psoas muscle.

#### 2.2.4. Endoscopic findings

**Figure 5.**
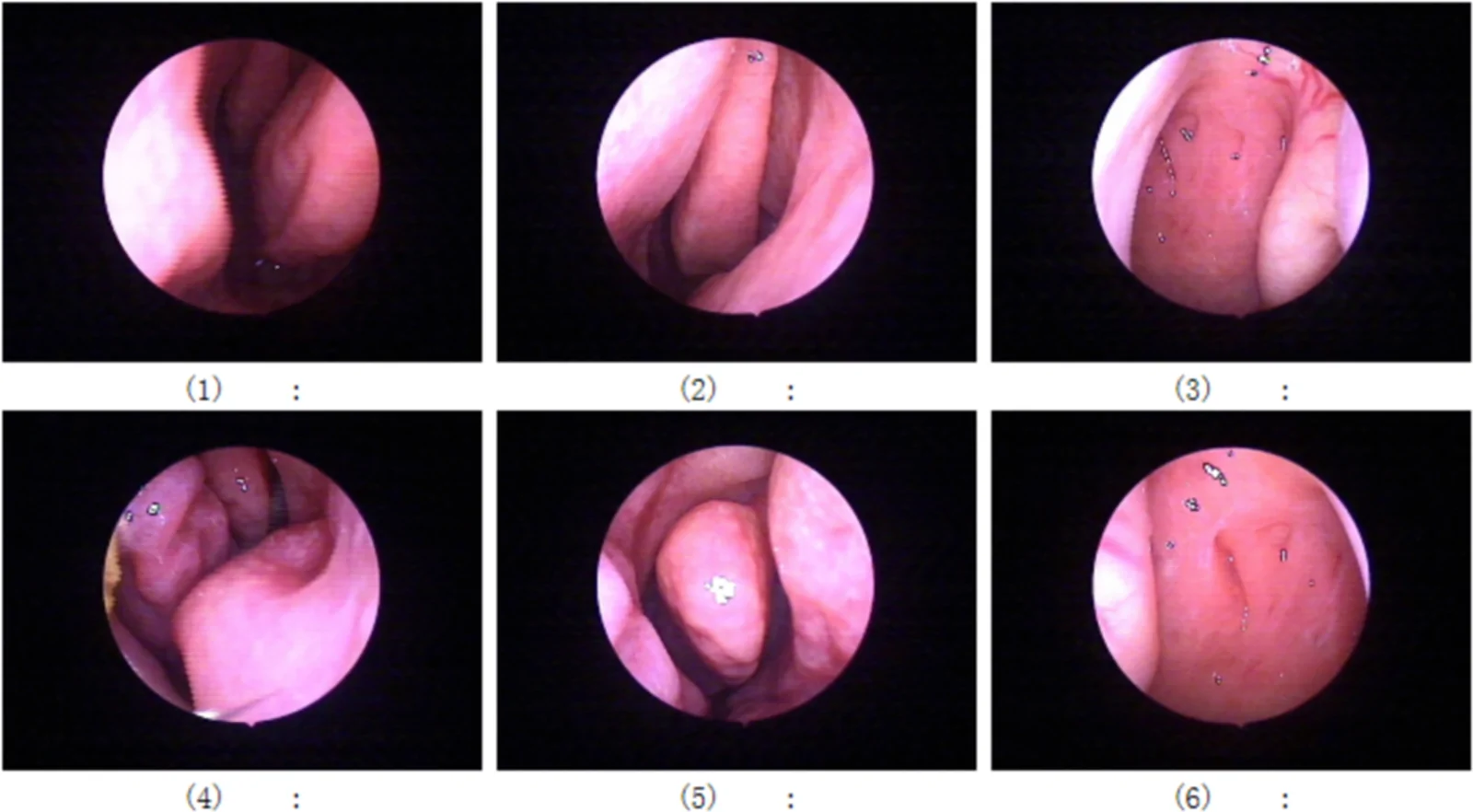
Nasopharyngoscopy of the patient.

**Figure 6.**
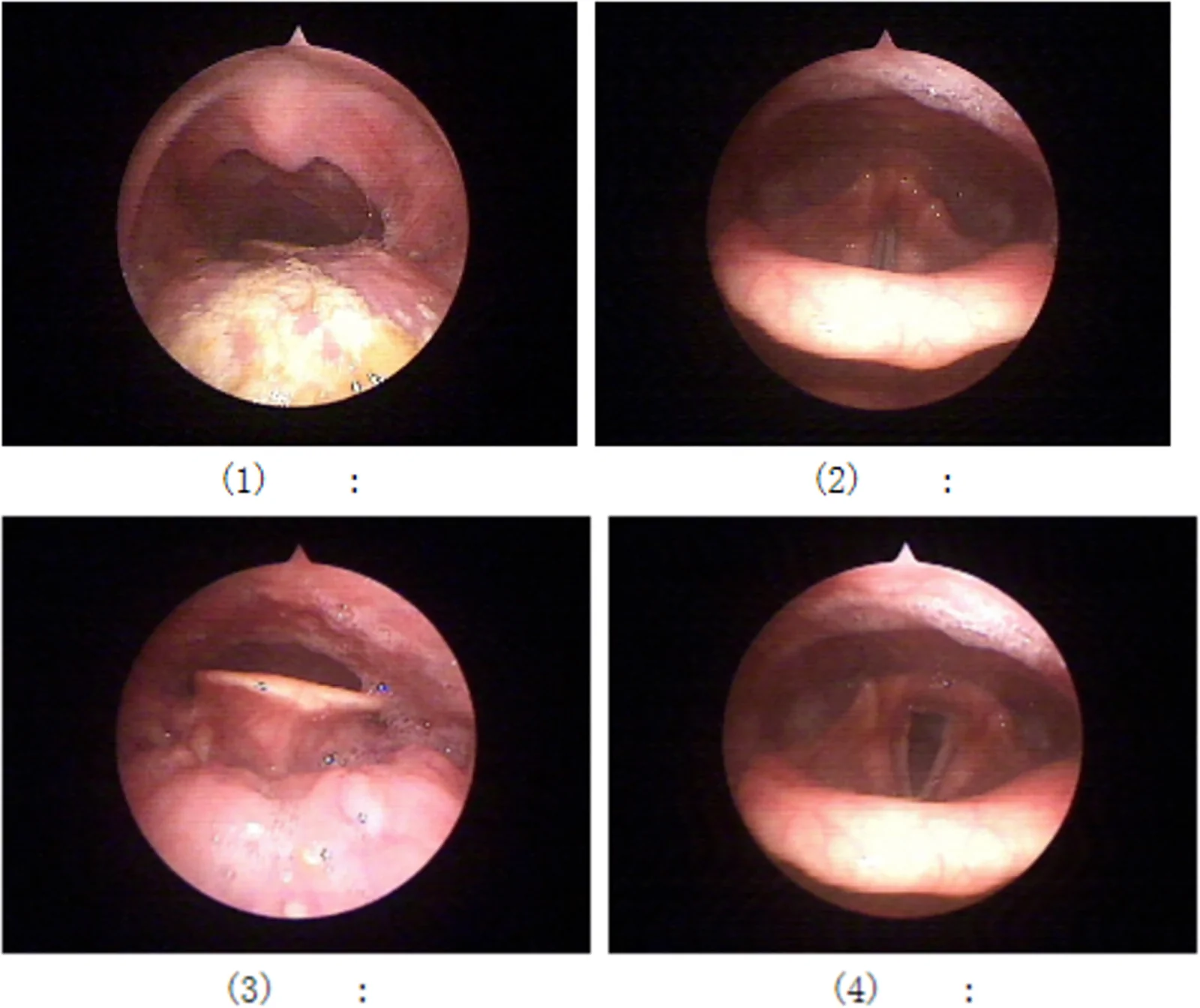
Laryngoscopy of the patient.

**Figure 7.**
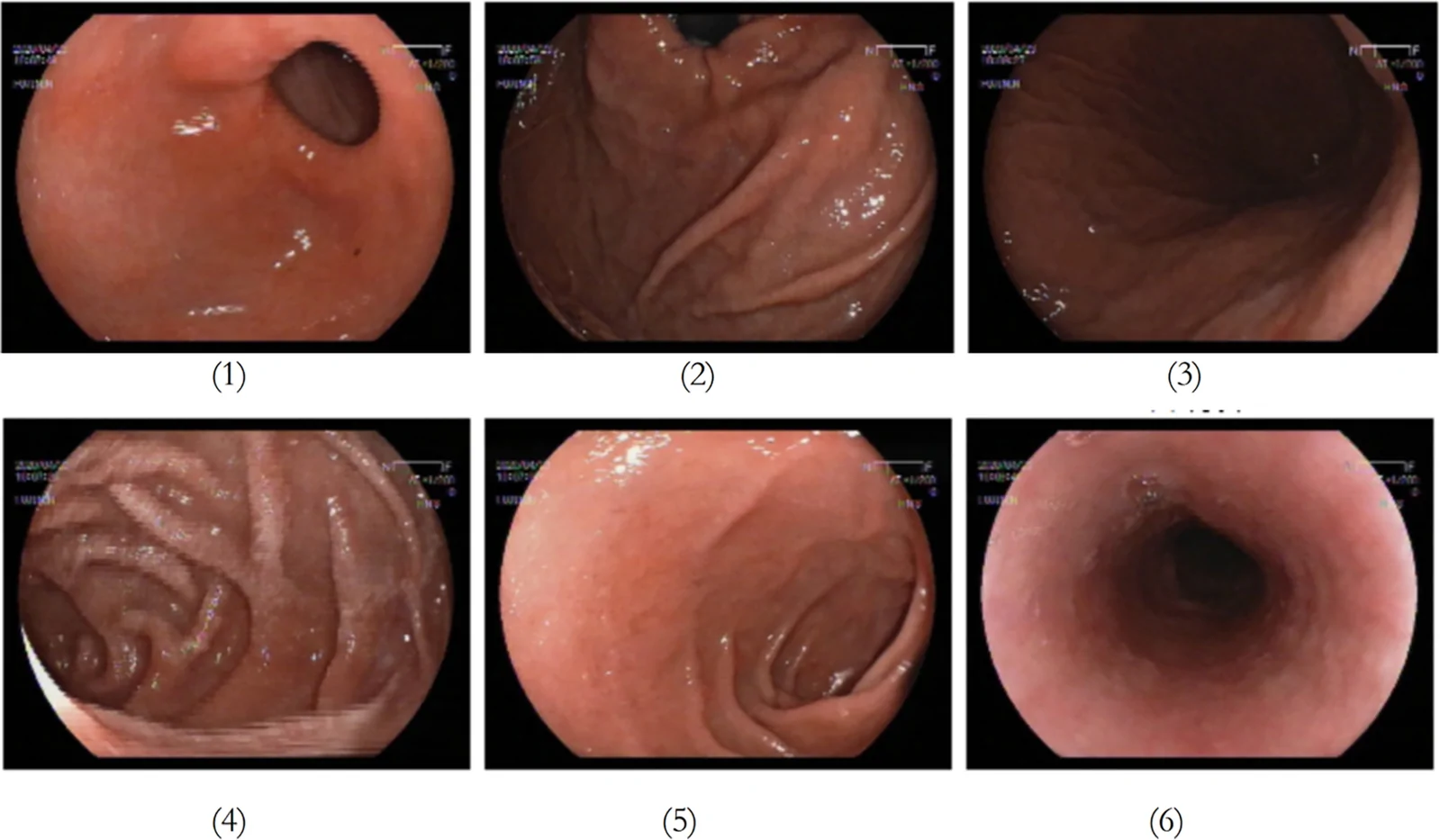
Esophagoscopy of the patient.

Nasopharyngoscopy(Fig. 5): Nasopharyngeal mucosal thickening; right vocal cord paralysis. Laryngoscopy (Fig. 6): No significant mass at the tongue base, chronic hyperemia of laryngopharyngeal mucosa, good epiglottic elevation, smooth surface, bilateral piriform sinuses symmetric with no obvious fluid or mass. Esophagoscopy (Fig. 7): No thickening or mass of the esophageal mucosa was found.

#### 2.2.5. Pathology and molecular diagnosis

On May 18, 2020, a biopsy of the mass (routine pathology) revealed the following immunohistochemistry (IHC) results: CK(−), TTF-1(−), CD20(+), CD3(−), KI67(>90%+), c-myc(90%+), P53(90%+), CD99(focal+), P63(focal weak+), syn(−), CgA(−), CD56(−), s-100(−), SOX-10(−), EBER(−). On May 20, 2020, the final pathology (combined IHC and fluorescence in situ hybridization [FISH] results) indicated high-grade B-cell lymphoma with MYC and BCL6 rearrangements. IHC results were as follows: CD20(+), CD3(−), KI67(>90%+), c-myc(90%+), bcl-2(−), CD10(−), bcl-6(+), P53(90%+), CD99(focal+), P63(focal weak+), cyclin D1(−), MUM1(−), TdT(−), EBER(−).

### 2.3. Final diagnosis, interventions, and outcome

Final diagnosis: High-grade B-cell lymphoma. On April 9, 2020, the patient developed dysphagia with a choking sensation, mild dysarthria, and intermittent muscle twitching of variable duration in all 4 limbs. He reported no ptosis or diplopia. The symptoms were persistent, aggravated by activity, and slightly relieved by rest. Over the preceding 2 months, he had experienced an unintentional weight loss of 5 kg but did not seek medical attention. He was admitted to our hospital on April 16, 2020, where swallowing rehabilitation training was initiated, along with clopidogrel for antiplatelet therapy and rosuvastatin to stabilize plaques. On May 12, 2020, a tender mass was noted in the right submandibular region, and a biopsy was arranged. Concurrently, a therapeutic trial of prednisone, pyridostigmine, and mecobalamin was started. However, on May 17, 2020, the patient developed a low‑grade fever (38.1°C), and pyridostigmine was discontinued. On May 20, 2020, based on immunohistochemical and FISH results, the diagnosis of high-grade B-cell lymphoma was confirmed, and systemic chemotherapy with the R-CHOP regimen (rituximab, cyclophosphamide, doxorubicin, vincristine, and prednisone) was initiated.

### 2.4. Follow-up

At the 2-month follow-up, the patient showed further weight loss, extensive extranodal and multi-organ involvement, indicating a poor prognosis.

## 3. Discussion

### 3.1. Diagnostic insights: from “bulbar palsy + Tapia syndrome” to high-grade B-cell lymphoma

The patient presented with dysphagia and mild dysarthria. Physical examination revealed a reduced right gag reflex, rightward tongue deviation, tongue atrophy and fasciculations, wasting of the bilateral interosseous and thenar muscles, unclear articulation, along with hyperactive deep tendon reflexes in all 4 limbs and positive upper limb pathological signs. EMG showed varying degrees of neurogenic changes in muscles innervated by bilateral C5-T1 spinal cord segments and early neurogenic changes in the left sternocleidomastoid muscle. These findings were highly suggestive of motor neuron disease. However, the patient also had right tongue atrophy and right vocal cord paralysis, indicating combined involvement of the X and XII cranial nerves, which fits the clinical features of Tapia syndrome (ipsilateral recurrent laryngeal branch of the vagus nerve and hypoglossal nerve palsy).^[1]^ The etiologies of Tapia syndrome are diverse and can be categorized into 3 main groups: mechanical injury, infectious factors, and malignant tumors.^[2]^ Most cases are a rare complication of surgery or anesthesia. The mechanism may involve compression of pharyngeal tissues by the laryngoscope, compression by the endotracheal tube cuff, or nerve traction from head and neck hyperextension or rotation.^[2]^ In 2014, Cantalupo et al reported the first lymphoma-associated Tapia syndrome. This typically indicates a peripheral lesion in the parapharyngeal space, similar to our case.^[2]^ Notably, the disease progressed rapidly, a feature that contrasts with the indolent course typical of motor neuron disease. Compared with classic amyotrophic lateral sclerosis, lymphoma-related cases often deteriorate within weeks to months and may present with sensory disturbances, autonomic dysfunction, or encephalopathy, suggesting involvement beyond the motor system alone.^[3,4]^ Importantly, the EMG abnormalities in this patient were confined to the cervical enlargement (C5-T1) and bulbar region (sternocleidomastoid muscle), while the lower limbs and thoracic paraspinal muscles were normal. Such localized neurogenic damage is relatively uncommon in motor neuron disease, which usually follows a more widespread, progressive, and cross-segmental pattern.^[3,4]^ We therefore suspected that the neurogenic damage could be attributed to lymphoma infiltration of anterior horn cells, nerve roots, or cranial nerves. The patient’s neostigmine test and repetitive nerve stimulation were both negative, excluding myasthenia gravis. Importantly, initial laryngoscopy and esophagoscopy were negative for tumors, but the patient had concurrent systemic symptoms (weight loss and splenomegaly). Thus, in patients with progressive bulbar palsy and Tapia syndrome, especially when accompanied by systemic manifestations, even negative routine endoscopy should prompt consideration of lymphoma, and timely imaging and biopsy are warranted to avoid misdiagnosis.

### 3.2. B symptoms are a group of systemic symptoms with important clinical significance in patients with lymphoma

B symptoms are a group of systemic manifestations with important clinical significance in patients with lymphoma. Their classic definition includes 3 core indicators: unexplained fever (body temperature > 38°C lasting more than 3 days), night sweats (profuse sweating during sleep that soaks clothing or bedsheets), and weight loss (unexplained loss of >10% of body weight within 6 months).^[5]^ In our patient, although fever and night sweats were absent early on, he developed a low-grade fever (38.1°C), rapid weight loss (5 kg), splenomegaly, elevated inflammatory markers (high-sensitivity c-reactive protein and erythrocyte sedimentation rate), and mild anemia one month after admission, which together suggested a tumor‑related systemic reaction. These systemic manifestations, combined with unexplained neurological deficits (such as bulbar palsy, dysarthria, tongue atrophy, and fasciculations in this case), should prompt early evaluation with PET-CT, contrast-enhanced imaging of suspected extranodal sites, and biopsy to avoid delayed diagnosis of lymphoma. Brain magnetic resonance imaging revealed soft tissue thickening and abnormal signals in the nasopharynx, longus capitis muscles, clivus, and anterior foramen magnum – areas often overlooked but anatomically close to the skull base.^[6]^ Such imaging abnormalities, rather than being dismissed as inflammatory or artifactual, should prompt strong consideration of lymphoma, nasopharyngeal carcinoma, or skull base metastases.^[7,8]^ When lymphoma invades the skull base, it often spreads diffusely along skull base foramina and intermuscular spaces because of abundant lymphoid tissue and the absence of capsular restriction, rather than forming a well-defined mass.^[9,10]^ In this case, subsequent PET-CT showed hypermetabolic lesions in the aforementioned regions with extensive involvement of the skull base bone and adjacent muscles. Thus, this case offers 2 important clinical lessons. First, early-stage lymphoma can present with atypical symptoms and without superficial lymphadenopathy; unexplained multiple neurological symptoms, B symptoms, and elevated inflammatory markers should never be ignored. Second, attention should be paid to early imaging findings that may serve as easily overlooked “alert signals.” Breaking out of the “preconceived” diagnostic framework of neurological diseases and paying attention to these atypical but critical clues is essential to avoid missing a diagnosis of lymphoma.

### 3.3. The etiology of carotid artery stenosis in cancer patients should be carefully evaluated

The patient in this case is a 46-year-old male with a long history of smoking (10 cigarettes/day). Although his lipid levels are normal, imaging studies have already shown atherosclerotic plaques in the abdominal aorta and peripheral arteries, suggesting the presence of systemic atherosclerosis. Atherosclerosis is the most common cause of carotid artery stenosis.^[11]^ It is worth noting that in healthy arteries without atherosclerosis, even when compressed externally by tumor masses, permanent severe stenosis may not occur readily because the arterial wall is elastic and has self‑regulatory capacity.^[12]^ In contrast, in arterial segments already affected by atherosclerotic lesions, lymphoma can invade cervical lymph nodes, the parapharyngeal space, and the soft tissues surrounding the carotid sheath. When enlarged lymph nodes or primary tumor tissue lie near the carotid bifurcation or the origin of the internal carotid artery, they may directly compress the arterial wall from the outside, which could lead to passive luminal narrowing^[13,14]^; the 2 factors may have an additive effect. In this case, PET‑CT showed hypermetabolic right cervical nodes and nasopharyngeal tumor infiltrating the medial pterygoid and longus capitis muscles, near the origin of the internal carotid artery, where ultrasound and CTA had already demonstrated atherosclerotic plaque with severe stenosis. We hypothesize that the lymphoma mass compressed the already less compliant artery, and the combination of atherosclerosis and external compression likely contributed to the severe stenosis. This patient has severe stenosis at the origin of the right internal carotid artery, which carries a potential risk of hypoperfusion-related or embolic stroke. This risk is particularly high during the cancer-related hypercoagulable state or when receiving systemic anti-tumor therapy, as hemodynamic fluctuations could trigger cerebral infarction. If successful neurointerventional treatment were performed, luminal patency could be immediately restored, reducing the peri-treatment risk of stroke and providing a safety guarantee for subsequent anti-tumor therapy. However, the patient has double-hit lymphoma with extensive systemic involvement, a poor prognosis, and a limited life expectancy. Post-procedure dual antiplatelet therapy would significantly increase the risk of fatal bleeding from the areas invaded by the tumor in the nasopharynx and skull base. Furthermore, there is a possibility that subsequent lymphoma soft-tissue infiltration elsewhere could externally compress large arteries, causing vascular stenosis. Therefore, a cautious evaluation is required.

## 4. Conclusion

This case presented initially with bulbar palsy and mild dysarthria, highly suggestive of motor neuron disease, but was ultimately confirmed as high-grade B-cell lymphoma with extensive involvement of the skull base, cranial nerves, anterior horn of the spinal cord, and nerve roots. This diagnostic process reveals 3 important clinical insights: First, Tapia syndrome (combined ipsilateral palsy of cranial nerves X and XII) together with localized neurogenic damage involving the cervical enlargement and bulbar region, in patients with rapid progression and a negative neostigmine test, should raise suspicion for direct invasion of the nervous system by systemic tumors such as lymphoma. Second, pay attention to B symptoms. Short-term weight loss, elevated inflammatory markers, and atypical imaging abnormalities serve as a key alert for early diagnosis of lymphoma. Moving beyond a preconceived diagnostic framework of neurological diseases and focusing on the “systemic wasting-neuropathic syndrome” as well as subtle imaging findings is essential to avoid missed diagnosis. Third, carotid artery stenosis in cancer patients should not be simply attributed to atherosclerosis. When a lymphoma soft-tissue mass coexists with atherosclerotic plaque, an additive effect may occur, and interventional treatment requires careful evaluation.

## Acknowledgments

We would like to express our gratitude to the patient for granting permission to use the patient’s clinical data in this paper and for the publication of this research.

## Author contributions

**Conceptualization:** Han Luo, Bo Liu.

**Data curation:** Han Luo, Zengxia Zhao.

**Formal analysis:** Han Luo, Zengxia Zhao.

**Funding acquisition:** Bo Liu.

**Investigation:** Han Luo, Zengxia Zhao, Bo Liu.

**Methodology:** Zengxia Zhao, Bo Liu.

**Resources:** Zengxia Zhao, Bo Liu.

**Software:** Zengxia Zhao.

**Supervision:** Zengxia Zhao, Bo Liu.

**Validation:** Han Luo, Bo Liu.

**Visualization:** Han Luo, Bo Liu.

**Writing – original draft:** Han Luo.

**Writing – review & editing:** Bo Liu.
